# Editorial: Omics integration and network medicine to decipher human complex diseases

**DOI:** 10.3389/fgene.2023.1119967

**Published:** 2023-03-31

**Authors:** Mario Zanfardino, Giulia Babbi, Kazim Yalcin Arga, Katia Pane

**Affiliations:** ^1^ IRCCS Synlab SDN, Naples, Italy; ^2^ Biocomputing Group, Department of Pharmacy and Biotechnology, University of Bologna, Bologna, Italy; ^3^ Department of Bioengineering, Faculty of Engineering, Marmara University, Istanbul, Türkiye; ^4^ Genetic and Metabolic Diseases Research and Investigation Center, Marmara University, Istanbul, Türkiye

**Keywords:** omics, data integration, network medicine, machine learning, multiomics

Most complex human diseases are caused by the perturbations of multiple molecular events, connected through biological pathways, that impact on the organism leading to a disease phenotype. The recent advances in multiomics and network medicine can drive deeper insights into human pathobiology. Network medicine applies the principle of network theory and systems biology to dissect complex cellular networks. However, to yield precision medicine there is a need for computation approaches encompassing multi-layered data integration, genome-wide studies (epigenomics, genomics, and proteomics) and data analysis, including machine learning (ML) algorithms. For this Research Topic, we sought high-quality original articles and reviews, providing novel insights into the domain of complex human diseases, such as cardiovascular diseases, oncology, neurological disorders, and metabolic diseases. The final Research Topic collected 8 articles addressing genome-wide association studies (GWAS); comprehensive analysis of disease signatures and pan-cancer analysis adopting multiomics approaches; regulatory networks among circRNA-miRNA-mRNA; and ML applications for candidate diagnostic and prognostic biomarkers.

The huge amount of data generated by GWAS needs to be analyzed to annotate causal variants, unravelling the underlying biological mechanisms that cause disease phenotypes. Amongst the plethora of post-GWAS methods available, Pérez-Granado et al. address the problem of variant interpretation using GWAS and expression quantitative trait loci (eQTL) data on the major depression (MD). They carried out a benchmark analysis using two approaches: fine-mapping and colocalization methods, to identify variants associated with a biological effect and their target genes. Their work has highlighted the challenges of post-GWAS analysis for understanding disease pathology, drug target prioritization, and biomarker discovery.

There is a continuous need to identify deregulated genes involved in complex diseases such as cancer. Yuan et al. performed a pan-cancer analysis of a poorly characterized family of genes: *FAM83* (Family with sequence similarity 83). They explored the transcriptomic profiling of the *FAM83* family by using TCGA cohorts for 33 cancer types and immunohistochemical data using the Human Protein Atlas. They related *FAM83* alterations and phenotypic variables, such as differential expression in TNM stages, to predict cancer progression using the UCSC Xena website, and related gene expression levels with tumor microenvironment and stemness scores across cancers using the ESTIMATE algorithm.

In another pan-cancer study, Liu and Wang systematically explored the Homologous Recombination Factor With OB-Fold (*HROB*) gene expression in many cancer types. They analyzed *HROB* expression using GTEx and TCGA transcriptome data. In addition, the authors show the application of the EPIC algorithm to associate *HROB* expression to the infiltration level of cancer-associated fibroblasts (CAFs) and CD8^+^ T cells; and performed survival and stemness analyses to sustain the hypothesis of *HROB* as a pan-cancer marker. Thereby, both pan-cancer studies showed the importance of large public cohorts and user-friendly bioinformatics resources for identifying candidate biomarkers.

As mentioned earlier, network-based approaches have a number of potential biological and clinical applications, from the identification of disease-related genes to defining better drug targets. The work by Kan et al. investigated the regulatory network underlying the Tetratology of Fallot (TOF), a congenital heart malformation. They constructed a triple regulatory network based on aberrantly expressed hub genes and their regulatory circRNAs and microRNAs, through multiple target prediction tools. Additionally, they identified seven hub genes involved in oxygen impairment, *via* protein-protein interaction (PPI) networks and functional genomics. This study revealed a potential regulatory axis, including competing endogenous RNAs as candidate diagnostic biomarkers for poorly-studied cardiac disease.

In another study, Wang et al. used four microarray datasets to identify circulating gene signatures of pulmonary arterial hypertension (PAH). They detected a number of upregulated, differentially expressed genes (DEGs) showing association with several KEGG pathways, thereby revealing 13 master transcriptional regulators linked with genes in PAH. Moreover, they also analyzed the interaction of hub proteins and FDA-approved drugs with significant binding affinity. Their findings could provide an interesting basis for the development of new diagnostic and treatment strategies for PAH.

In recent years, the so-called cellular heterogeneity, *i.e.,* the diversity of regulatory programs characterizing each cell type and state, has emerged as another fundamental aspect to be addressed in understanding the basis of disease.

From a multiomics perspective, single-cell sequencing can contribute to dissecting cellular heterogeneity. Following this idea, Gao et al., combined single-cell RNA sequencing, gene expression, and methylation data for studying the *CRIP1* regulation networks in patients with Acute Myeloid Leukemia (AML). They shed light onto genetic, immunological, and epigenetic perturbations, such as hyperexpression of *CRIP1* in t (8; 21) AML (including M0–M7 subtypes), its abnormal activation by the TNFα-NFκB signaling pathway, as well as immune infiltrate dysfunctions that may affect patients’ prognosis. They also found that the DNA methylation level of CRIP1 was negatively correlated with CRIP1expression.

The results of multiomics data integration are the starting point for developing predictive models for biomarker identification and diagnosis. In this scenario, ML algorithms offer new insights compared with classical statistical methods. This Research Topic includes two innovative studies using multiple ML algorithms to develop predictive models for the diagnosis of diabetic foot ulcer (DFU) and obstructive sleep apnea (OSA).

Wang et al. employed weighted gene co-expression network analysis to identify gene modules consisting of DFU-associated genes, and presented a set of ferroptosis genes that can distinguish DFU patients from healthy controls. They constructed an accurate DFU prediction model on this gene cluster, combining four popular ML algorithms (LASSO, SVM-RFE, Boruta and XGBoost), and identified *MAPK3* and *MAFG* as the leading components that could also be potential therapeutic targets for DFU patients. Similarly, Zhu et al. used three machine-learning algorithms (SVM-RFE, RF, and ANN) to develop an RNA-level predictive tool to estimate risk for OSA, and the therapeutic response to continuous positive airway pressure (CPAP) treatment. They also identified possible immunologic mechanisms associated with the development of OSA and showed that patients at high risk for OSA tended to have elevated levels of inflammation that could be reduced by CPAP treatment. Both studies confirmed the improved predictive power of using multiple ML algorithms and the critical role of immunity in the development and treatment of these diseases.

